# Antibiotic prescription and clinical management of common infections among general practitioners in Latvia, Lithuania, and Sweden: a pilot survey with a simple protocol

**DOI:** 10.1007/s10096-017-3141-2

**Published:** 2017-12-07

**Authors:** Uga Dumpis, Annika Hahlin, Sonata Varvuolyte, Stephan Stenmark, Sarmīte Veide, Rolanda Valinteliene, Asta Jurkeviciene, Johan Struwe

**Affiliations:** 1 Pauls Stradins University Hospital, University of Latvia, Riga, Latvia; 2 Strama Stockholm, Stockholm, Sweden; 3 Vilnius University, Lithuanian Society of General Practitioners, Vilnius, Lithuania; 4 Strama Västerbotten, Umeå, Sweden; 5 Latvian Family Physicians’ Association, Riga, Latvia; 6 Klaipeda University, Institute of Hygiene, Klaipeda, Lithuania; 7 Public Health Agency of Sweden, Stockholm, Sweden

## Abstract

Comparative information on diagnosis-related antibiotic prescribing patterns are scarce from primary care within and between countries. To describe and compare antibiotic prescription and routine management of infections in primary care in Latvia (LV), Lithuania (LT) and two study sites in Sweden (SE), a cross-sectional observational study on patients who consulted due to sypmtoms compatible with infection was undetraken. Infection and treatment was detected and recorded by physicians only. Data was collected from altogether 8786 consecutive patients with infections in the three countries. Although the overall proportion of patients receiving an antibiotic prescription was similar in all three countries (LV and LT 42%, SE 38%), there were differences in the rate of prescription between the countries depending on the respective diagnoses. While penicillins dominated among prescriptions (LV 58%, LT 67%, SE 70%), phenoxymethylpenicillin was most commonly prescribed in Sweden (57% of all penicillins), while it was amoxicillin with or without clavulanic acid in Latvia (99%) and Lithuania (85%) respectively. Pivmecillinam and flucloxacillin, which accounted for 29% of penicillins in Sweden, were available neither in Latvia nor in Lithuania. The applied methodology was simple, and provided useful information on differences in treatment of common infections in ambulatory care in the absence of available computerized diagnosis–prescription data. Despite some limitations, the method can be used for assessment of intention to treat and compliance to treatment guidelines and benchmarking locally, nationally, or internationally, just as the point prevalence surveys (PPS) protocols have been used in hospitals all over Europe.

## Introduction

Antibiotic use is a major factor in driving antibacterial resistance [1–3]. In addition, unnecessary use of antibiotics entails an increased risk of side-effects [4] as well as additional costs [5].

Outpatient prescriptions account for the majority of antibiotic use in humans in Europe [6]. Still, information on the reason for prescribing is difficult to obtain. Most international studies comparing ambulatory consumption of antibiotics have been based on aggregated data from prescription databases or from wholesale figures. According to these sources, the consumption of antibiotics and antimicrobial resistance rates of community-acquired pathogens in Latvia, Lithuania, and Sweden have been among the lowest in the European countries [6, 7].

The aim of this study was to describe and compare the routine antibiotic prescribing practices and clinical management of common community-acquired infections in primary care in three countries around the Baltic by using a simple 1-week diagnosis–prescription study protocol. A similar approach has previously been successfully used nationally in Finland, Sweden, and Latvia [8–10]. Management of respiratory tract infections has also been studied internationally in the Happy Audit projects [11, 12].

## Methods

**Fig. 1:**
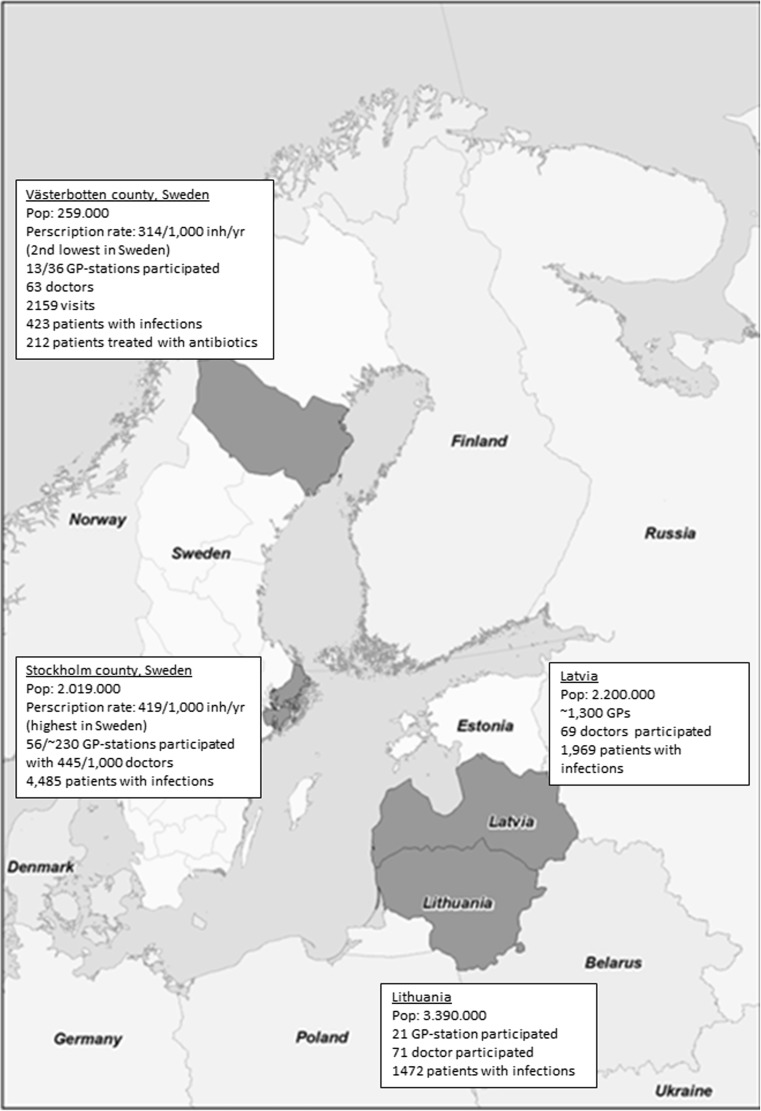
Study sites in a point prevalence survey of clinical management for infections in three countries around the Baltic Sea

In order to assess management and antibiotic prescription during 1 week in GP practices, we used a cross-sectional 1-week point prevalence approach. GPs were asked to record data for each patient consulting with clinical signs of a possible infection during 1 week from November 22 (7 AM) to November 28 (7 PM) in 2010. Centers and doctors in each participating region were recruited through convenience sampling in Latvia, Lithuania, and the regions of Stockholm and Västerbotten in Sweden. Participation was voluntary and did not involve financial incentives. A general overview of some background data is given in Fig. 1.

In Latvia, family physicians are self-employed and usually located individually. In Lithuania, some family physicians work in group practices, some in policlinics, and a very small number own single practices. In this study, we had family physicians representing all types of practices. In Sweden, most GP practices have between three and ten GPs. All participating doctors completed the protocols on their own; there was no cross-validation.

In Latvia, an e-mail signed by the head of the association and chief investigator containing an invitation to participate in the study was sent to all selected GPs through the mailing list registered at the Latvian Family Physicians Association.

In Lithuania, invitations were sent via e-mail and mail to primary health care centers and family physicians directly as well. Institute of Hygiene and Lithuanian Society of General Practitioners created the information–invitation form for study, and invited all family doctors who expressed willingness to participat to an introductory seminar.

In Stockholm county, Sweden, invitations were sent to the responsible doctor at every GP practise and also to each individual doctor according to a mailing list kept by Strama Stockholm. In the invitation mail, each practise was asked to provide a contact person who then was invited to a seminar.

In Västerbotten county, Sweden, Strama sent an invitation to participate to all responsible doctors via the county council’s intranet and also via e-mail.

Inclusion criteria were all clinic and home visits and phone consultations by patients of all ages with symptoms of a possible infection. We included all new cases and failures (relapses, complications). Exclusion criteria were: recovery patient consultation for follow-up.

Anonymized data on demographic factors, diagnosis based on a pre-defined list, laboratory investigations, X-ray and — in the case of antibiotic prescription — substance, dose, dosing interval, and route of administration were recorded in a data collection form that was prepared for this study and translated into the appropriate national language. The total number of all patients consulting for any diagnosis at each practice during the study week was also collected. The completed data collection forms were mailed back to the site coordinator and data entered into a data base.

The ATC classification for antibiotics was used [13].

In Latvia, the study was approved by Pauls Stradins Clinical University Hospital Development Fund Ethical Committee as part of the National Research Programme BIOMEDICINE. In accordance with this decision, consent forms were not necessary since patients’ and doctors’ information was not collected. In Sweden, follow-up of antibiotic prescriptions is part of ongoing quality assurance and patient safety programs, and ethical approval is not needed for collection of anonymized data. In Lithuania, regulation was similar to that in Sweden and written consent from patient was not required.

The data was processed using EpiInfo 7 and SPSS 20 software.

## Results

Altogether, data was obtained on 8786 infections (LV, 1970; LT, 1522; SE, 4889). Swedish data from Stockholm and Västerbotten were merged during analysis as Västerbotten recruited a much smaller number of patients and there were no major differences in practice between the two sites.

Some characteristics for patients, use of diagnostics, and prescription of antibiotics are compared between the countries in Table 1.

**Table 1:** General information on patients, use of diagnostics, and treatment in a point prevalence survey of ambulatory treatment and antibiotic prescription for infections in three countries around the Baltic Sea

Variable	Latvia	Lithuania	Sweden
Mean age (all patients, years)	20 (STd 20)	20 (STd 19)	31 (STd 25)
Gender (all patients)	Female 55% (95% CI, 53%–7%)	Female 53% (95% CI, 50%–55%)	Female 40% (95% CI, 39%–2%)
Mean age in patients who received an antibiotic	26.9 (STd 22.4)	24.7 (STd 21.3)	37.2 (STd-26.1)
Gender in patients who received an antibiotic	Female 58% (95% CI, 54%– 61%)	Female 52% (95% CI, 48%– 56%)	Female 35% (95% CI, 33%–37%)
Mean duration of symptoms before the visit (days)	6.4 (STd 11)	7.3 (STd 16)	11 (STd 17)
Proportion receving an antibiotic prescription (%)	42% (95% CI, 40%–45%)	42% (95% CI, 39%–44%)	38% (95% CI, 37%–40%)
CRP test performed (% of *all* cases with infection)	7% (95% CI, 6%–8%)	27% (95% CI, 25–29%)	32% (95% CI, 31%–33%)
Nitritis test performed in cases with uncomplicated urinary tract infection	57% (95% CI, 44%–70%)	68% (95% CI, 53%–83%)	70% 95% CI, 65%–75%)
Strep A test performed (% of pharyngotonsillitis cases)	11% (95% CI, 7%–15%)	0.3% (95% CI, 0.3%–0.9%)	74% (95% CI, 70%–78%)
X-ray performed (% of pneumonia cases)	3% (95% CI, 0.4%–6%)	74% (95% CI, 62%–85%)	1.4% (95% CI, 0.6%–3.4%)

*STd* Standard deviation, *CI* Confidence interval

The overall proportion of patients receiving antibiotic prescription was in the same order of magnitude in all three countries. Patients in SE were older, and had symptoms longer before seeking GPs. Patients younger than 20 years constituted the majority of all patients receiving antibiotic prescriptions in Latvia (51%) and Lithuania (53%), while in Sweden this age group constituted 33%. In contrast, in Sweden a higher proportion of prescriptions were to persons older than 50 years (32%), compared to 19% in Latvia and 13% in Lithuania.

Strep A test for pharyngotonsilitis was more frequently used in SE, while X-ray for pneumonia diagnosis was mainly used in patients in LT. Dipstick/nitritis tests were used for more than half of the patients with uncomplicated UTI, most frequently in SE.

**Fig. 2:**
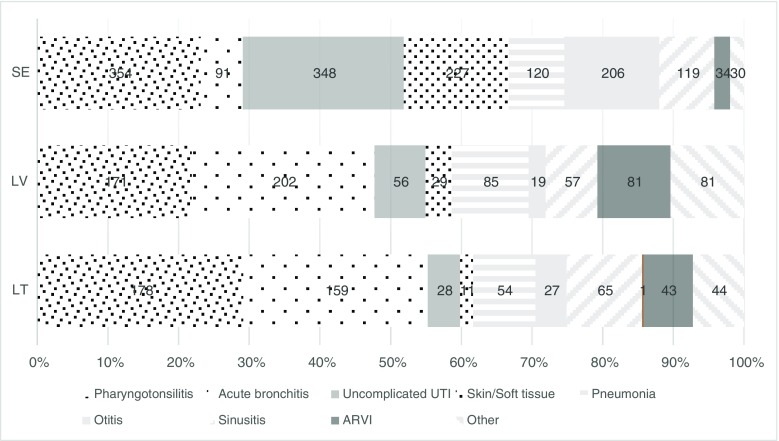
Distribution (%) between major groups of infections treated with antibiotics in Sweden (*SE*), Latvia (*LV*) and Lithuania (*LT*). *ARVI* — acute respiratory virus infection, not specified. *UTI* — urinary tract infection

Figure 2 illustrates the most common diagnoses treated with antibiotics.

**Fig. 3:**
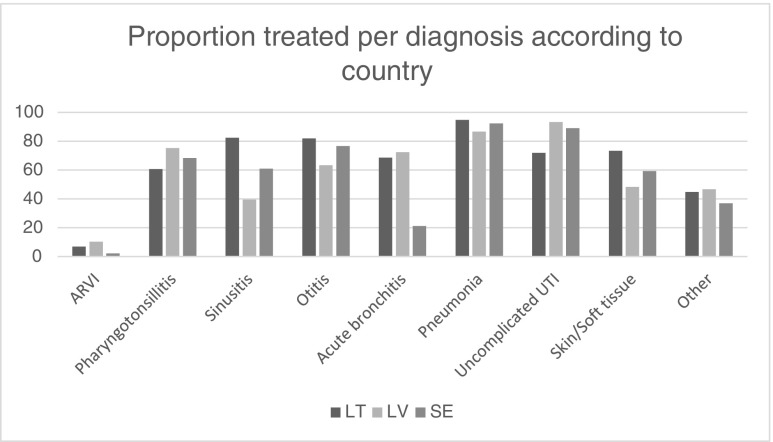
Proportion of patients per diagnosis treated with antibiotics in Sweden (*SE*), Latvia (*LV*) and Lithuania (*LT*). *ARVI* — acute respiratory virus infections, not specified. *UTI* — urinary tract infection

Figure 3 shows that the most obvious difference in antibiotic prescriptions per diagnosis was seen in treatment for acute bronchitis, which was significantly lower (*p* < 0.05) in Sweden at 21% (95% CI, 16.2%–23.8%), versus 68% (95% CI, 62.5%–73,5%) in LT and 72% (95% CI, 66.2–77.8%) in LV. The difference in the proportion of antibiotic prescriptions in patients diagnosed with sinusitis was also significantly lower in Latvia at 40% (95% CI, 32%–48%), compared to Lithuania (82%; 95% CI, 73.5%–90.5%) and Sweden SE (61%; 95% CI, 54.2%–67.9%), *p* < 0.05).

**Fig. 4:**
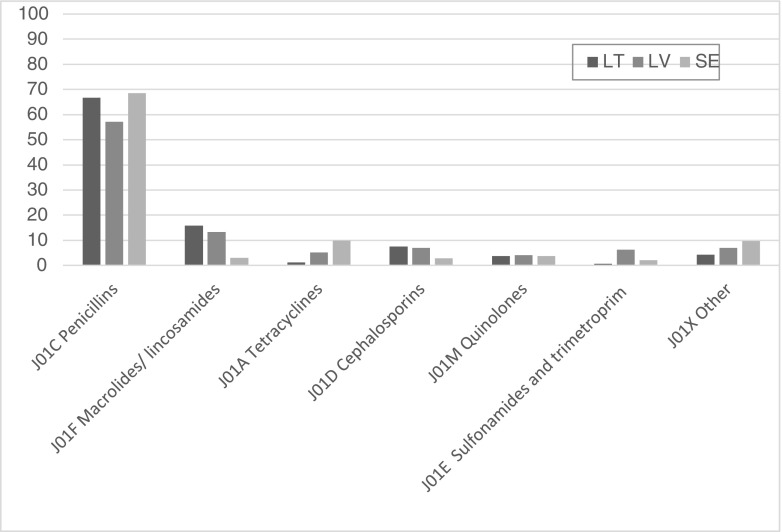
Distribution of prescribed antibiotic classes in Lithuania (*LT*), Latvia (*LV*), and Sweden (*SE*)

Figure 4 shows that the most frequently prescribed antibiotic group in all countries were penicillins at 66% (LV 58%, LT 67%, SE 70%). Other main groups in LV and LT were macrolides/ lincosamides (LV 9.6%, LT 15%, SE 3.1%.), and in SE tetracyclines (LV 5.2%, LT 1.2%, SE 9.8%).

The most frequently prescribed penicillins in LV and LT were amoxicillin, 72% and 60% respectively, and amoxicillin/clavulanate, 27% and 26% respectively. In SE it was phenoxymethylpenicillin (57%) and pivmecillinam (17%). Flucloxacillin, which accounted for 12% of penicillins in Sweden, was available neither in Latvia nor in Lithuania.

Table 2 shows the precriptions for some of the most frequent diagnoses. Major differences were that penicillin V was predominantly prescribed for otitis and pharyngotonsillitis in Sweden, while it was amoxicillin with or without enzyme inhibitor in Latvia and Lithuania. For pneumonia, penicillin V, amoxicillin, and doxycyclin dominated in Sweden, while macrolides, cephalosporins, and “other” were comparatively more frequently used in Latvia and Lithuania.

**Table 2:** Proportion of prescription of different antibiotics (%) for most commonly treated diagnoses in primary care in Latvia, Lithuania and Sweden

	Latvia	Lithuania	Sweden
Otitis	*n* = 19	*n* = 27	*n* = 206
- Penicillin V	0	0	74
- Amoxicillin	37	33	17
- Amoxicillin + enzyme inhibitor	32	37	0.5
- Macrolides	10	3.7	2.5
- Other	21	26.3	6.4
Pharyngotonsillitis	*n* = 171	*n* = 178	*n* = 354
- Penicillin V	2.9	21	89
- Amoxicilllin	60	48	2.3
- Macrolides	4.1	3.3	1.1
- Amoxicillin + enzyme inhibitor	16	9	0.3
- Other	17	18.7	7.3
Pneumonia	*n* = 85	*n* = 54	*n* = 120
- Penicillin V	0	0	52
- Amoxicillin	16	5.6	13
- Amoxicillin + enzyme inhibitor	29	24	2.5
- Macrolides	27	32	4.1
- Doxycycline	4.7	0	27
- Cefuroxime	9.4	20.4	0
- Other	13.9	18	1.4
Uncomplicated UTI	*n* = 56	*n* = 28	*n* = 348
- Pivmecillinam	0	0	54.9
- Amoxicillin	16	3.6	0
- Nitrofurantoin	36	54	32
- Fluoroquinolones	23	25	2.3
- Trimethroprim/ Sulfamethoxazole	12	7.1	0
- Other	13	10.3	10.8

## Discussion

We describe the first international diagnosis–prescription study in general practice covering all infections based on an easy-to-use protocol where demographic information on patients and data on diagnostics, treatment, and indications for antibiotic prescription were collected [11, 12]. In general, findings were quite similar in Latvia and Lithuania compared to in Sweden, with regard to both prescriptions and to the use of point-of-care tests. This could be explained by different health care systems and the existence of national guidelines for diagnosis and treatment of around ten different infections common in primary care in Sweden [11]. These have been extensively communicated by local Strama groups and drug therapeutic committees, and feed-back of compliance and open benchmarking based on individual prescription data are routinely used. In contrast, national treatment guidelines are available neither in Latvia nor in Lithuania. International studies on antibiotic prescription in general practice are scarce, but have been published on respiratory tract infections in the Happy Audit projects [12, 13].

Symptoms of upper respiratory tract infection were the most common indication for seeing GPs and for prescribing an antibiotic, as found in other studies [8–10]. Frequent prescription of antibiotics for self-limited conditions in Latvia and Lithuania, such as acute bronchitis, was of particular concern. This suggests a need for national treatment guidelines in Latvia and Lithuania and strategies for implementation of these.

The use of the Strep A test for pharyngotonsillitis was, in contrast to Sweden, very limited in Latvia and Lithuania, as found in Happy Audit [12]. Implementation of Strep A according to Centor criteria could guide clinicians to select the patients whom might benefit from antibiotic treatment.

The main group of antibiotics prescribed in all countries were the penicillins. However, the same preference of amoxicillin/clavulanate over phenoxymetylpenicillin as found before intervention in the Happy Audit projects [12, 13] was seen in Latvia and Lithuania. A limiting factor is that phenoxymetylpenicillin is hardly available due to the registration and marketing situation in Latvia and Lithuania, and is also considerably more expensive than amoxicillin.

Fluoroquinolones are widely used for treatment of community-acquired urinary tract infection (UTI) despite the worldwide increase of quinolone-resistant *Escherichia coli* strains [14, 15]. According to the Swedish treatment guidelines, pivmecillinam, which was available neither in Latvia nor in Lithuania, along with nitrofurantoin are the first line drugs for uncomplicated UTI. Likewise, flucloxacillin, which is the drug of choice for staphylococcal skin- and soft-tissue infections in Sweden, was not available in Latvia and Lithuania. Instead, more expensive antibiotics with broader spectrum further driving resistance are used. This further supports the need for national guidelines and interventions in these countries.

Different patterns for seeking the GP probably contributes to some differences seen in the study. Higher age and longer duration before seeking care in Sweden may suggest that children with viral respiratory infections are given symptomatic treatment at home to a higher extent, while instead they see a doctor in Latvia or in Lithuania. This may also explain why patients with uncomplicated urinary tract or skin/soft tissue infections accounted for a greater proportion of antibiotic prescriptions in Sweden. Overall, a majority treated with antibiotics were small children, as previously reported [16].

Despite the fact that the vast majority of antibiotics are prescribed in the ambulatory setting [17], there are no computerized medical record systems in Latvia and Lithuania, and data on prescription linked to diagnosis is quite difficult to extract despite the IT-based systems in Sweden. Thus, manual descriptive prevalence studies are probably still the easiest way to obtain information, as shown in the numerous hospital point prevalence studies (PPS) performed around Europe and elsewhere [18, 19]. International comparisons allow identification of areas for improvement and help in designing interventions.

The purpose of this pilot survey was to test the international applicability of the protocol and to get a base-line indication of similarities and differences in routine clinical management of common infections in general practice. Weaknesses were that no diagnostic criteria or other guidelines were provided, and that the GPs who participated could be biased towards being more motivated and interested in the field than average. In addition, active data collection itself may influence the prescription habits and accuracy of recorded diagnoses.

In conclusion, a simple manual diagnosis–prescription cross-sectional survey provided useful information on antibiotic prescription in ambulatory care in three contries around the Baltic Sea, and pointed at several areas for improvement. National treatment guidelines should be developed and educational interventions implemented in Latvia and Lithuania. Older effective antibiotics, such as phenoxymetylpenicillin, pivmecillinam, and cloxacillin should be equally accessible across the EU. Despite some limitations, the methodology can be used to support antimicrobial stewardship in general practice and benchmarking locally, nationally, or internationally.
